# Uncovering Different Masking Factors on Wrist Skin Temperature Rhythm in Free-Living Subjects

**DOI:** 10.1371/journal.pone.0061142

**Published:** 2013-04-05

**Authors:** Antonio Martinez-Nicolas, Elisabet Ortiz-Tudela, Maria Angeles Rol, Juan Antonio Madrid

**Affiliations:** Chronobiology Laboratory, Department of Physiology, College of Biology, University of Murcia, Murcia, Spain; Kent State University, United States of America

## Abstract

Most circadian rhythms are controlled by a major pacemaker located in the hypothalamic suprachiasmatic nucleus. Some of these rhythms, called marker rhythms, serve to characterize the timing of the internal temporal order. However, these variables are susceptible to masking effects as the result of activity, body position, light exposure, environmental temperature and sleep. Recently, wrist skin temperature (WT) has been proposed as a new index for evaluating circadian system status. In light of previous evidence suggesting the important relationship between WT and core body temperature regulation, the aim of this work was to purify the WT pattern in order to obtain its endogenous rhythm with the application of multiple demasking procedures. To this end, 103 subjects (18–24 years old) were recruited and their WT, activity, body position, light exposure, environmental temperature and sleep were recorded under free-living conditions for 1 week. WT demasking by categories or intercepts was applied to simulate a “constant routine” protocol (awakening, dim light, recumbent position, low activity and warm environmental temperature). Although the overall circadian pattern of WT was similar regardless of the masking effects, its amplitude was the rhythmic parameter most affected by environmental conditions. The acrophase and mesor were determined to be the most robust parameters for characterizing this rhythm. In addition, a circadian modulation of the masking effect was found for each masking variable. WT rhythm exhibits a strong endogenous component, despite the existence of multiple external influences. This was evidenced by simultaneously eliminating the influence of activity, body position, light exposure, environmental temperature and sleep. We therefore propose that it could be considered a valuable and minimally-invasive means of recording circadian physiology in ambulatory conditions.

## Introduction

The circadian system is organized into a hierarchical network of structures that are responsible for the generation of circadian rhythms and their synchronization to environmental factors. This system includes a central pacemaker (the suprachiasmatic nucleus of the hypothalamus, SCN), several peripheral clocks, inputs and outputs, SCN pathways and the connections between them [1], [2].

Certain circadian outputs, known as circadian marker rhythms, are being used to assess the overall status of the circadian system. These marker rhythms are variables that can be used to characterize the timing of the internal temporal order. To be considered a circadian marker rhythm, the variable must be easily measurable over long periods of time, preferably using non-invasive methods. To date, the most widely used marker rhythms are core body temperature (CBT), and plasma or salivary melatonin [3], [4].

The circadian rhythm of CBT is determined by changes in heat production and heat loss [5], which is delayed with respect to heat gain [6]. Therefore, distal skin temperature (DST) is very important in the regulation of CBT as evidence has recently suggested. Changes in CBT are preceded by opposite changes in DST or wrist temperature (WT) [7], [8]. DST is becoming more widely used because it is less invasive, more comfortable, easy to use and stable under a constant routine as reflected by an increasing body of literature [8]–[17]. However, like CBT, DST is subject to many environmental and physiological influences that mask its rhythms. These include physical activity, body position, light exposure, environmental temperature and sleep [5], [17]–[23].

Both variables (CBT and WT) are the result of two sets of influences: one endogenous, directly driven by the SCN [23]–[25], and the other exogenous, exerting a masking effect and superimposed onto the endogenous factors. Activity and sleep are the masking factors that are most frequently studied [26]–[28]. However, other masking factors have been reported to include environmental temperature [21], body position [23], environmental light [18], [20] and even menstrual cycle, which affects mainly CBT, but not DST [29].

Several methods have been described to suppress masking effects on the CBT rhythm. The most widely used method is a protocol that reduces the masking effect by submitting the subjects to a constant routine. Under this routine, sleep is forbidden, and the subject is kept in bed in a semirecumbent body position, with constant mental activity and dividing food and drink into frequent small portions of constant composition, evenly distributed throughout both day and night [30], [31]. However, this protocol introduces its own masking effects, as it is stressful, unpleasant and quite unsuitable for repeated assessments [26], [28], [32]. Moreover, these experimental conditions with constant light and temperature produce negative consequences for the subject's physiology and are very artificial, as people tend to live according to rhythmic conditions [33]. Another disadvantage of this protocol is that it does not provide information about the effects of the environment on circadian rhythms [28]. A second method used to obtain the endogenous timing is forced desynchronization. In this case, subjects are exposed to 20-h or 28-h days, which are beyond entrainment limits. As a result, the circadian system reveals its endogenous timing, with similar results for both conditions (20-h and 28-h days) [34]. However, this method fails to show the organism's internal timing under normal living conditions (24-h day).

To eliminate masking effects in free-living subjects, most authors use mathematical tools. The first method developed is referred to as purification by categories, and it has mainly been applied to activity and heart rate. According to this method, CBT values are classified by activity levels, which are divided into categories and then used to analyze CBT data. The advantage of this approach is that by selecting temperature values corresponding to the lowest categories, researchers can simulate a “constant routine” approach to compensate for these masking factors [26], [27], [35], [36].

A second mathematical procedure to eliminate masking under free-living conditions is a method called purification by intercepts, or its more complicated version ANCOVA [37]. This method uses a regression analysis to calculate the core temperature that would correspond to zero activity or a low heart rate for each time bin, obtaining an approximate “constant routine” temperature curve [37], [38].

Although some authors did not achieve good results when applying demasking techniques in forced desynchrony protocols [39], it is a reliable option to obtain the endogenous pattern under normal free-living conditions [27], [37]. These methodologies have been tested to unmask the core body temperature rhythm, and the results obtained from each method are quite similar [27]. Moreover, the data purified by mathematical procedures and the “constant routine” protocol yield similar results [40]. However, to date, these procedures have not been applied to unmask variables other than CBT.

In light of the fact that the DST rhythm is subject to several environmental and behavioral masking effects, and since an increasing number of papers are focused on DST, the aim of this work was to obtain, for the first time, the endogenous circadian pattern of this rhythm by mathematical procedures for simultaneously removing the masking effects of light exposure, environmental temperature, sleep, activity and body position and to determine the influence of these masking variables on the WT rhythm.

## Materials and Methods

### Subjects

For the present study, 103 undergraduate student volunteers (48 men and 55 women, 18–24 years old) residing in Murcia, Spain (latitude 38° 01′ N) were recruited. All the recordings were made in November. The overall mean (±SEM) environmental temperature was 16.3±0.5°C and the natural photoperiod was between sunrise at 07:28–07:49 and sunset at 17:51–18:09 (Data obtained from the University of Murcia weather station [41]). Participants were instructed to complete a sleep diary designed by the Chronobiology Lab at the University of Murcia and were encouraged to maintain their habitual life style. The diary compiled information regarding sleep periods, time the subject went to bed and the time he or she got up. The chronotype of all participants was assessed using the morningness-eveningness questionnaire [42].

The study abides by the bioethical principles set out by the Declaration of Helsinki. Data from the volunteers were included in a database and were protected according to Spanish Law 15/1999 from 13 September. All participants received the appropriate information about the characteristics of the study and signed an informed consent form before their inclusion in the study [43]. The study was approved by the Ethical Review Committee from the University of Murcia. No research was conducted outside our country of residence.

### Wrist temperature measurement

All subjects wore a Thermochron iButton DS1921H (Maxim Integrated Products, Sunnyvale, California, USA) that measured their wrist skin temperature with a precision of ±0.125°C. This temperature sensor was placed on the wrist of the non-dominant hand over the radial artery and isolated from the environmental temperature by a double-sided cotton sport wrist band, as previously described [8], [12], [13]. Temperature sensors were programmed to sample every 10 minutes over the course of an entire week.

### Body position and activity monitoring

Body position and activity rhythms were assessed every 30 seconds using a HOBO Pendant G Acceleration Data Logger UA-004-64 actimeter (Onset Computer, Bourne, Massachusetts, USA) positioned on the non-dominant arm by means of a sport band. These data were then averaged for 10-minute intervals, allowing for WT comparisons. The manufacturing specifications and the method used to obtain these variables have already been described in a previous work [12]. Activity was measured as the rate of change in degrees per minute, and body position was calculated as the angle between the axis of the acelerometer parallel to the humerous bone and the horizontal plane.

### Environmental temperature and light exposure recording

In addition, all subjects were required to wear a HOBO Pendant Temperature/Light Data Logger UA-002-64 (Onset Computer, Bourne, Massachusetts, USA) on a necklace close to eye level during waketime and to put it on the bedside table during the sleep time to record environmental temperature and light exposure. Manufacturing specifications, memory, spectrum and accuracy were as described in a previous work [13]. This device records light intensity at regular intervals that have been previously programmed (in this experiment, every 30 seconds). These data were also averaged over 10-minute intervals to obtain the same sampling frequency as for WT.

### Data analysis

WT data were filtered in order to eliminate artifacts such as those produced by temporarily removing the temperature sensor. To that end, the interquartile distance (from Q1 to Q4) was calculated and each datum whose rate of change with respect to the previous value was higher than the interquartile distance was eliminated [8], [44]. Sleep-wake information was converted into binary values by assigning a value of 1 when the subjects declared they were asleep and 0 when awake, as has been previously described. Sleep probability indicates the percentage of individuals asleep at any given time, as already described [8], [12], [13].

For purposes of comparison, WT was purified for activity by means of two standard methods: categories and intercepts. The first procedure based on categories, as described by Waterhouse et al. [27]. To this end, individual activity was divided into terciles, and the values corresponding to the lower activity category (lower tercile of activity for each subject) were averaged for hourly intervals, and then the corresponding synchronous temperature values were used to reconstruct the WT mean rhythm.

The second procedure to demask WT was the purification by intercepts method [36]. This procedure was performed using hourly intervals of activity and its corresponding temperature, which were linearly correlated. The extrapolated temperature associated with zero activity was then assigned to the initial time-point of the hourly interval. The procedure was repeated to account for all 24 hours.

We also propose an extension to the purification by categories method originally reported by Waterhouse et al. [27] to determine the masking effect produced on WT by each individual variable. The environmental temperature categories used were cool (12–19°C), warm (19–26°C) and hot (26–33°C). In the case of activity, the categories were low (up to 33% activity for each individual subject, as previously described), medium (from 33% to 66%) and high (above 66%). To categorize body position, three intervals were considered: lying down (0–30°), leaning (30–60°) and standing (60–90°). Light exposure was divided in two categories: dim light (less than 10 lux) and non-dim light (more than 10 lux). Finally, the sleep variable was classified as sleep or wake state. Separate temperature curves for each category and variable (environmental temperature, light exposure, activity, body position and sleep) were obtained. Only those time points that included more than 15 subjects were considered.

To characterize the WT endogenous component under a protocol simulating a “constant routine”, we performed a multiple demasking procedure by categories or intercepts, simultaneously considering the masking variables of light exposure, environmental temperature, sleep, activity and body position. For purification by categories, individual WT data were selected to calculate an hourly interval waveform only when the following conditions were met: wake period, dim light (less than 10 lux), warm environmental temperature (19–26°C, a range matching that used in constant routine protocols, according to Graw et al. [45] or Jasper et al. [46]), body position between 0 and 30° (according to Cajochen et al. [47]), and low activity (less than 33%). For the purification by intercepts, we performed a stepwise multiple regression for each subject, for one-hour periods to identify the intercepts for WT rhythm and thus to educe WT values under conditions of wakefulness, recumbent body position, absence of activity, dim light and three different environmental temperatures (15, 20 and 25°C). The equation applied to each of the 24 hour periods for this demasking procedure is as follows:
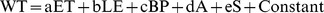



Where ET: Environmental Temperature; LE: Light Exposure; BP: Body Position; A: Activity; S: Sleep. The polynomial coefficients of each variable are a, b, c, d and e. These coefficients were represented per time point in order to establish the time-dependent influence of each masking variable on WT. In addition, the constant is also represented, and corresponds to the extrapolation to an environmental temperature of 0°C, 0 lux, 0 grades of position, 0 activity and no sleep.

To test whether the demasked WT rhythm allows for detecting differences in circadian phase for human chronotypes, two subgroups of 12 people each (belonging to higher and lower decile) were selected using the Horne-Östberg morningness-eveningness questionnaire [42]. A Student's t-test was performed to compare morning and evening types, before and after the demasking procedure.

The WT, ET, LE, BP, A and S rhythms were characterized using cosinor analysis. Rhythm parameters estimated from the cosinor procedure included its mesor (24 h rhythm-adjusted mean of the cosine curve fitted to the data), amplitude (difference between the maximum and the cosine calculated mesor) and acrophase (peak of the fitted cosine curve). All data are expressed as a mean with a 95% confidence interval. This inferential statistical method also provides the percent rhythm (%V; percentage of overall variance attributed to the best fitted cosine curve with reference to total variability of experimental data made equal to 100%) and a probability or p value that indicates the statistical significance of the fitness of the cosine curve to the data (rhythm detection level) and it was determined using the integrated package for temporal series analysis “El Temps” (A. Díez-Noguera, Universitat de Barcelona, 1999). The data were processed using Microsoft Office Excel 2007. The remaining data are expressed as mean ± SEM.

## Results

**Figure 1:**
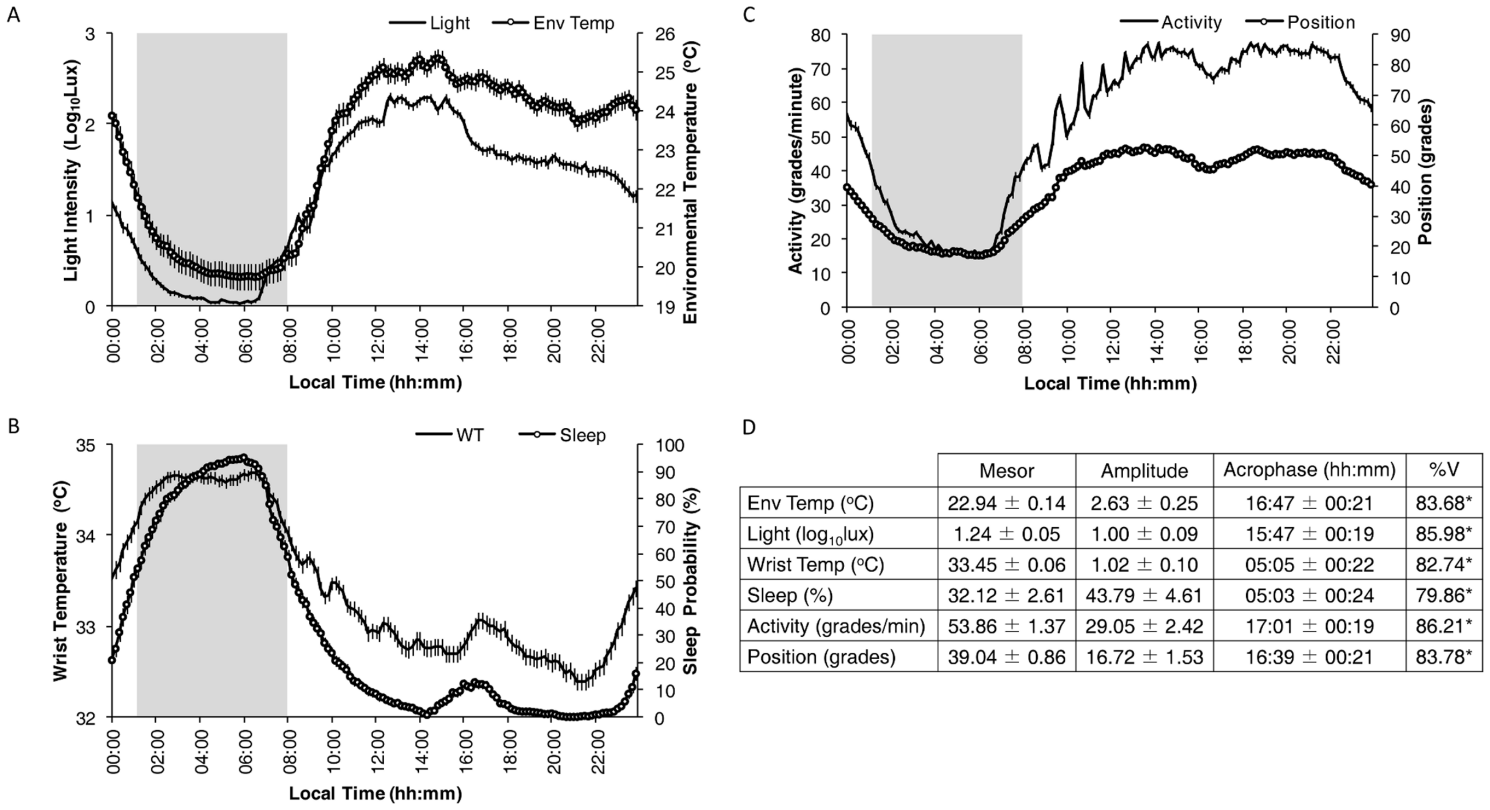
Study population mean-waveforms. Mean-waveforms for light exposure (Light) and environmental temperature (Env Temp) (A), wrist temperature (WT) and sleep (B), and activity and position (C). The shaded area shows the mean sleep period. All variables are expressed as mean ± SEM (n=103). The mean values (±95% Confidence Interval) for Mesor, Amplitude and Acrophase, as well as the %V as calculated by the cosinor analysis for the above-mentioned variables, are shown in D. * indicates p<0.001 according to the cosinor analysis.

The mean waveform of all recorded variables is shown in Figure 1. To facilitate the description of results, when more than 50% of volunteers were asleep, that period was considered the sleep period (01:10 h to 08:00 h), whereas when less than 50% were asleep, this was referred to as the wake period (08:10 h to 01:00 h). As can be observed in Figure 1A, the light exposure rhythm exhibited minimum mean values (7.95±1.59 lux) from 02:00 to 06:50 h, maximum mean values (162.19±1.12 lux) from 12:00 to 15:50 h, and reached a plateau of 38.02±1.12 lux from 17:00 to 22:50 h. The lowest mean values (20.16±0.32°C) of the environmental temperature rhythm coincided with the sleep period, while the highest mean values (24.08±0.24°C) occurred during the wake period. The minimum values for WT were observed from 20:10 h to 22:10 h (Figure 1B), coinciding with minimal sleep probability, a period known as the wake maintenance zone (32.48±0.09°C and 0.34±0.19% for WT and sleep probability, respectively). Both variables showed the highest values (82.22±1.90% for sleep probability and 34.54±0.07°C for WT) during the sleep period. In addition, a secondary peak in sleep probability (9.04±1.45%) was obtained at the postprandial time (15:00 to 17:50 h), with a slightly delayed (16:00 h to 18:50 h) increase in WT (32.90±0.09°C). As expected, activity and body position showed low, stable values during the sleep period (22.85±0.74°/min and 20.77±0.93°, respectively) and higher, more variable values (66.62±1.04°/minute and 46.56±1.04°, respectively) during the wake period. Again, a small decrease in both variables was observed coinciding with the postprandial dip.

The cosinor analysis of all rhythmic variables is shown in Figure 1D. A coincidence was detected between the acrophases of WT and sleep probability and the bathyphases (minimum value of the cosinor curve) of activity (05:01±00:19 h), body position (04:39±00:21 h) and environmental temperature (04:47±00:21 h). However, it should be noted that the light exposure bathyphase showed a slight phase advance (03:47±00:19 h) with respect to the other variables.

**Figure 2:**
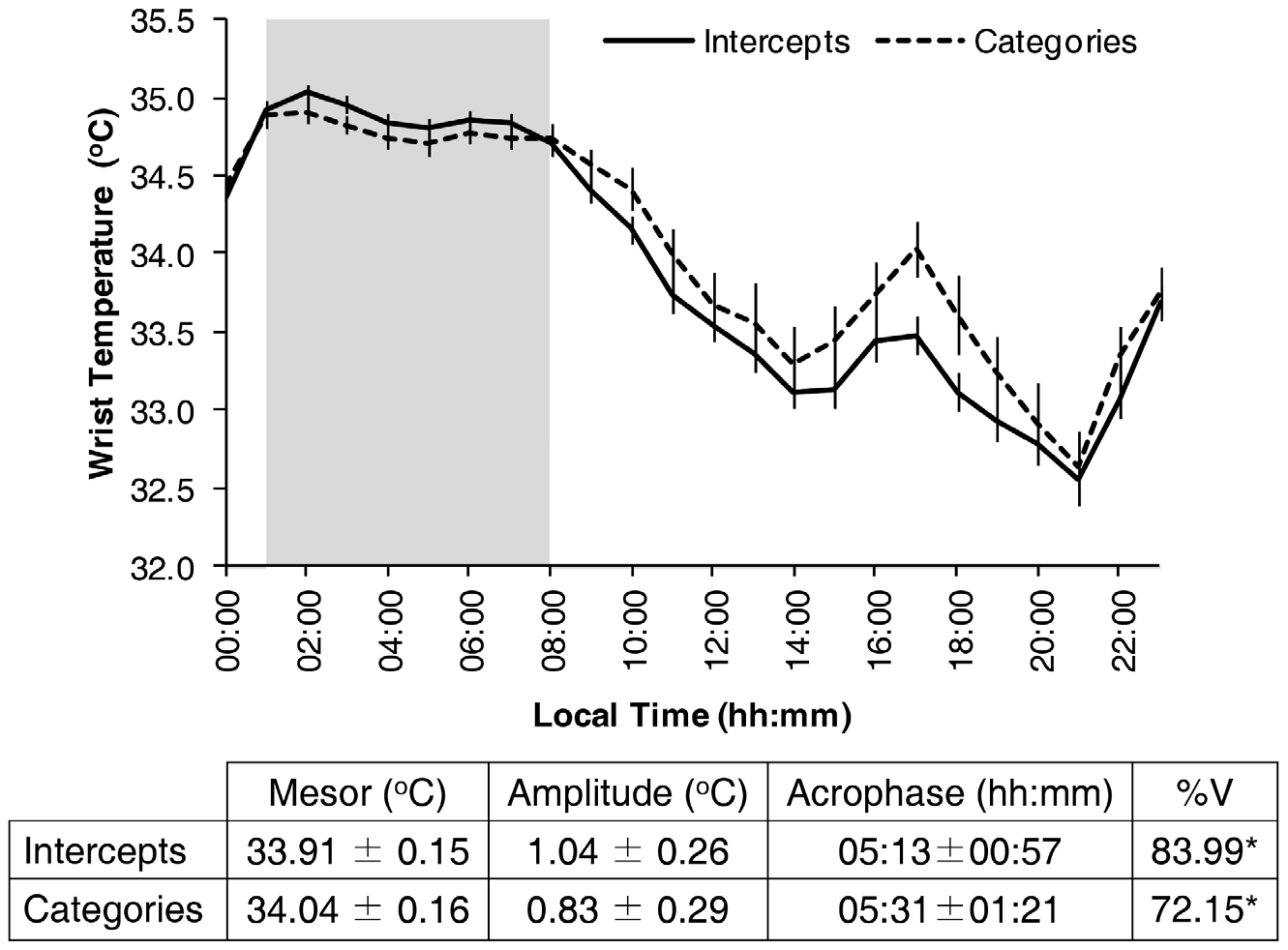
WT pattern purified for activity. Demasked WT pattern, expressed as mean ± SEM after application of the purification by intercepts or categories method (correcting for the effect of activity). The shaded area shows the mean sleep period. The table below the graph shows the corresponding Mesor, Amplitude and Acrophase as well as the %V for WT, demasked by means of the purification by categories or intercepts method, (data are expressed as Mean±95% Confidence Interval). * indicates p<0.001 according to the cosinor analysis.

The influence of activity on WT was determined by two demasking procedures: intercepts and categories (Figure 2). Both demasked curves present a roughly similar pattern, composed of three characteristic periods: high values during the sleep period (01:00 h to 08:00 h), a secondary postprandial peak (16:00 h to 18:00 h) and low values during the wake maintenance zone (20:00 h to 22:00 h). The two demasking procedures yielded significant rhythms (p<0.001) with no significant differences in mesor, amplitude or acrophase (Figure 2).

**Figure 3:**
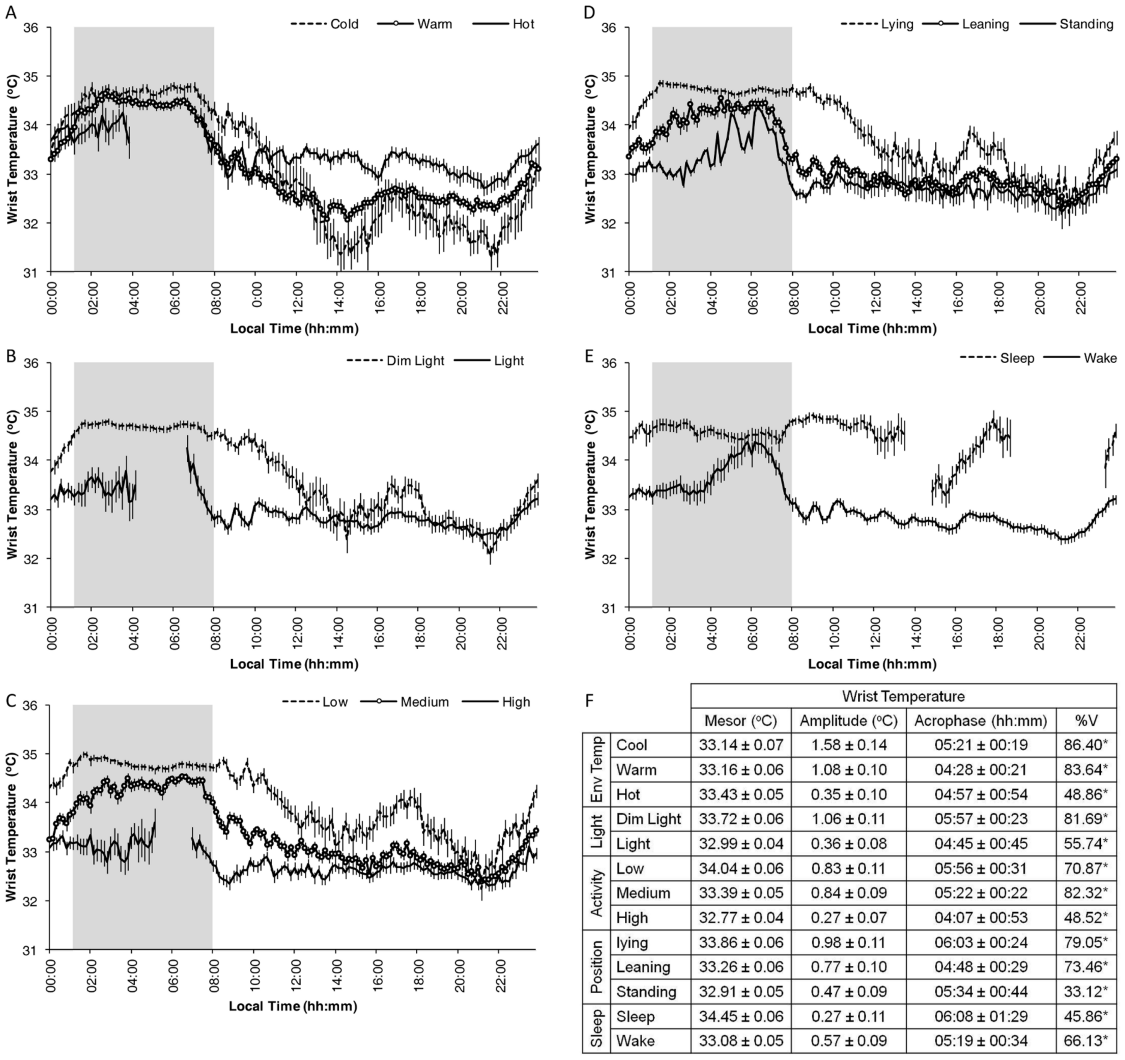
WT pattern purified for each studied masking variable. Demasked WT pattern, expressed as mean ± SEM, following the application of the purification by categories method according to environmental temperature level (A), light exposure (B), activity (C), position (D) and sleep status (E). The shaded area shows the mean sleep period. Note that in the case of the lowest level of activity, the same data set as for Figure 1 has been used, although in this case, with intervals of ten minutes instead of one hour. See the material and methods section for more details. The values for Mesor, Amplitude and Acrophase as well as the %V of each demasked wrist temperature pattern are expressed as Mean±95% Confidence Interval, and are included in F. * indicates p<0.001 according to the cosinor analysis.

The category procedure was used to determine the contribution of each individual variable to WT (Figure 3).

The contribution of environmental temperature to WT is shown in Figure 3A. Although the mesor of the WT rhythm remained unaffected, its amplitude was significantly reduced as the environmental temperature increased (Figure 3F, cosinor analysis), with increased wake time values and slightly decreased sleep time values. Non-significant differences in demasked WT pattern acrophases were observed among environmental temperature categories. In addition, the wake maintenance zone was present in all curves, although high environmental temperatures increased WT even in this zone.

The demasking to remove the effects of light exposure is shown in Figure 3B. WT shows higher values with dim light than with non-dim light, except during two short periods at noon and again in the wake maintenance zone. Non-dim light reduces both nocturnal WT and its postprandial increase and, therefore, the mesor and amplitude of the WT pattern (Figure 3F, cosinor analysis).

Demasking, thereby eliminating the effects of activity and body position are shown in Figures 3C and 3D, respectively. WT presents similar patterns despite activity level or body position (higher nighttime and lower daytime values, and the acrophase from 04:00 to 06:00 h). However, higher activity levels or positions flatten the WT pattern and reduce WT values, reducing its mesor, except in the wake maintenance zone, in which all activity and body position categories displayed similar WT values (Figure 3F, cosinor analysis).

Sleep demasking is shown in Figure 3E. In spite of the large number of subjects recruited, the WT sleep-demasked curve presents two periods with an insufficient number of sleeping subjects (from 13:40 to 14:50 h and 18:50 to 23:10 h). Sleep increases WT values regardless of the time when sleep occurs (increasing mesor values, Figure 3F), with the exception of around 06:00 h when sleep and wake curves rendered similar values.

**Figure 4:**
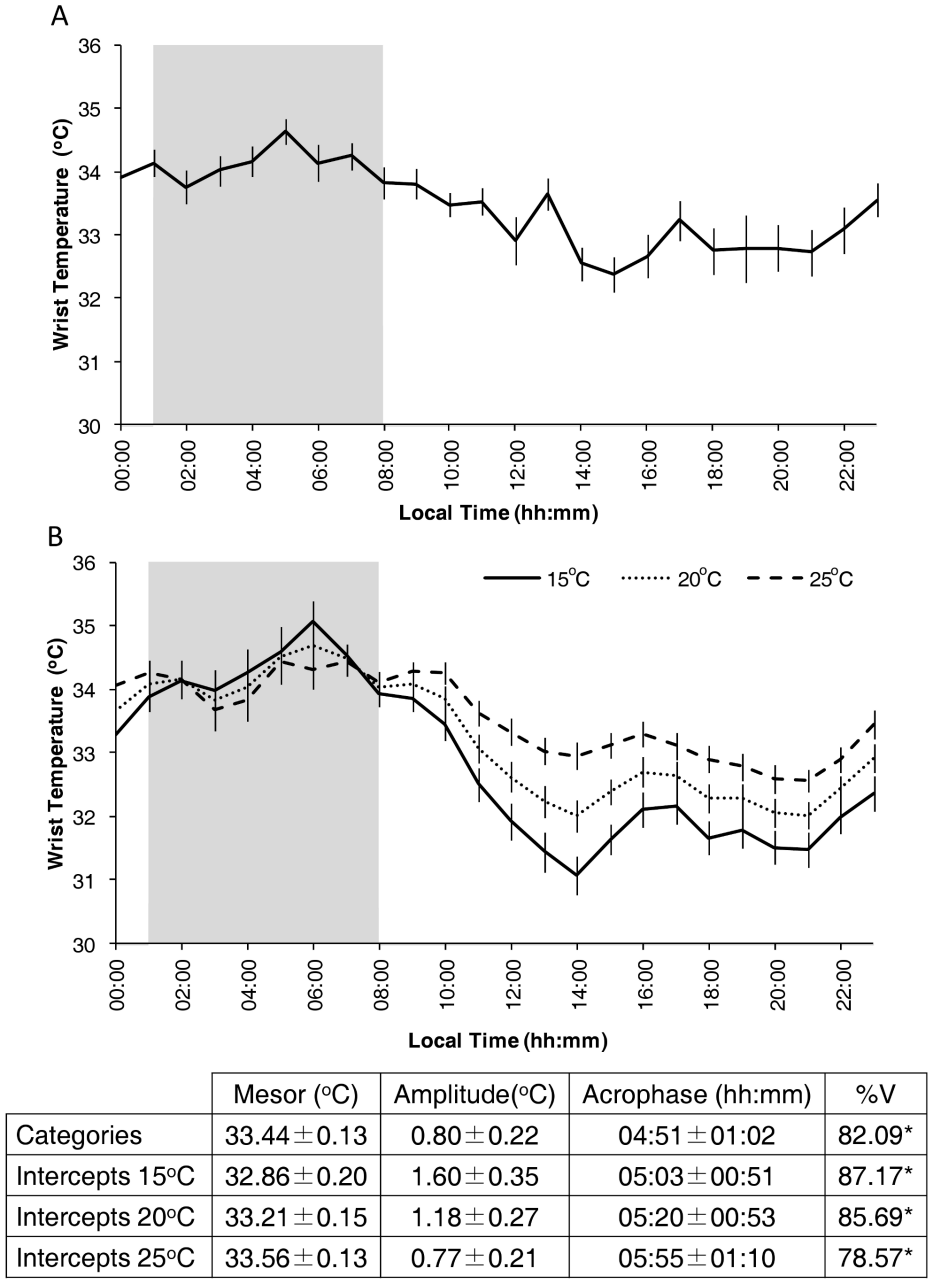
WT pattern after constant routine approach. Demasked WT waveforms obtained using the constant routine approach (see the material and methods section for details), employing either purification by categories (A) or by intercepts (B); in the latter case, three different environmental temperatures (15°, 20° and 25°C) are considered. The shaded area shows the mean sleep period. Data are expressed as Mean ± SEM. The values for Mesor, Amplitude and Acrophase as well as the %V of wrist temperature demasked by the simulated constant routine are expressed as Mean±95% Confidence Interval and are included at the bottom of the graph. * indicates p<0.001 according to the cosinor analysis.

Figure 4 shows the WT patterns obtained after mathematical simulation of the “constant routine” protocol based on either categories (Figure 4A) or stepwise multiple regression intercepts (Figure 4B). The effect and the modulation by masking factors were quantified by the polynomial coefficient itself. If modulation did not occur, a constant coefficient would be obtained over the 24 h period. The constant routine by categories method yielded a WT pattern similar to that obtained by the intercepts method at 20°C and 25°C (see also the cosinor analysis at the bottom of the graph. Interestingly, environmental temperature seems to exert an effect during the activity phase, but not during the rest phase.

When this mathematical simulation was applied to morning and evening subgroups, acrophases were stable before and after demasking (03:49±00:15 and 04:12±00:37 for morning type, and 05:50±00:35 and 06:23±00:33 for evening type, respectively) and their corresponding phase difference was maintained between types (p<0.05 for both raw and demasked data).

**Figure 5:**
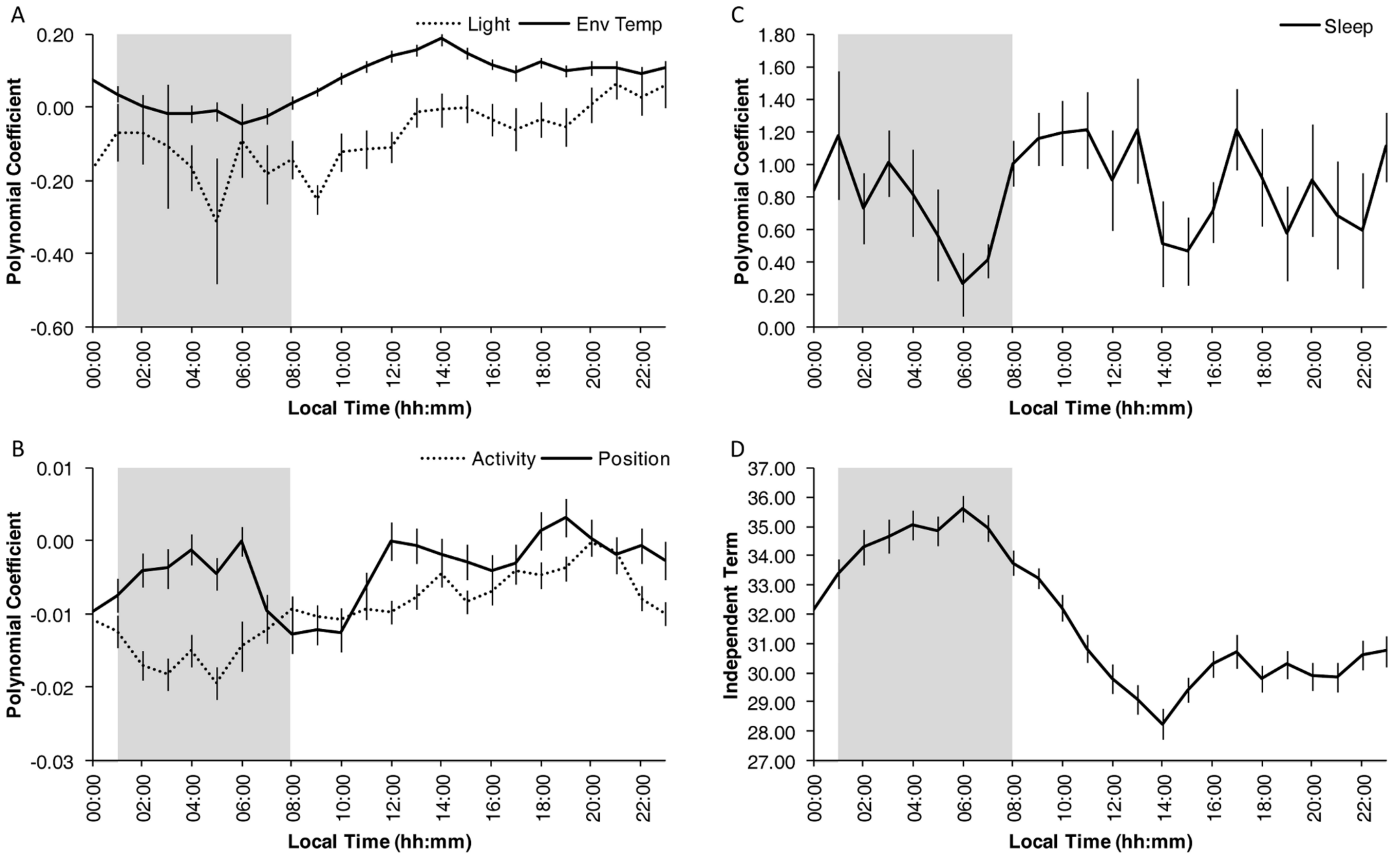
Circadian modulation of masking variables. Circadian modulation of the polynomial coefficients for the different masking variables: (A) light and environmental temperature (Env Temp), (B) activity and position and (C) sleep. The independent term for WT is represented in D. The shaded area shows the mean sleep period. Note that the independent term corresponds to an environmental temperature of 0°C. All values are expressed as Mean ± SEM. See the materials and methods section for details.

Circadian modulation of the masking effects induced by each variable is shown in Figure 5. The circadian pattern in light exposure masking indicates that exposure to high levels of light reduced WT from 00:00 to 12:00 h, but it had no effect from noon to midnight (Figure 5A). However, higher environmental temperature values failed to mask WT from 01:00 to 08:00 h, but they increased WT throughout the rest of the day (Figure 5A). The influence of body position was also modulated during the circadian cycle, with two main masking periods being evident around the usual times for going to bed (at midnight) and getting up in the morning (Figure 5B). When subjects lay down, their WT increased; on the other hand, when they got up, their WT decreased. In the case of activity, WT generally decreased, with the maximum influence exerted during the sleep period. It seemingly had no effect in the wake maintenance zone, however.

Sleep is related with higher WT values throughout the day, but this increase was lower at times when the volunteers were usually sleepy (in the postprandial zone and the maximum sleepiness zone around 06:00 h, as shown in Figure 5C).

When all these masking influences were removed by representing the polynomial independent term (that is, the WT pattern under the specific condition where the rest of variables are zero), WT exhibited a roughly sinusoidal pattern (Figure 5D), with its acrophase at 04:40±00:53, amplitude of 2.91±0.66°C and mesor of 31.82±0.37°C.

## Discussion

Our results show that despite the existence of multiple external influences on the wrist skin temperature rhythm, it exhibits a strong endogenous component which can be uncovered by using different demasking procedures to eliminate the influence of activity, body position, light exposure, environmental temperature and sleep. Although the overall circadian pattern is similar for both the masked and unmasked WT, there are changes in individual rhythmic parameters. Amplitude was most affected by environmental conditions, while the acrophase and mesor were the most stable and robust parameters for characterizing the circadian rhythm. These results suggest that the WT rhythm may prove to be valuable and minimally-invasive means of assessing circadian phase in ambulatory conditions, once further research determines appropriately consistent and accurate correlations with other well-established marker rhythms, for example dim light melatonin onset (DLMO).

To the best of our knowledge, this is the first time that demasking procedures have been applied to any variable other than CBT or that multiple masking factor influences (environmental temperature, light exposure, activity, sleep and body position) have been simultaneously removed by applying these mathematical techniques to a rhythmic variable.

Both demasking methods (intercepts and categories) produce similar WT mean waveforms and yield unmasked WT rhythms with characteristics similar to those of the raw WT circadian pattern reported in this paper and by others [8], [10], [12]. Thus we can conclude that the endogenous (unmasked) WT rhythm has the same three characteristic stages of the raw WT rhythm in subjects under free-living conditions [8] and for DST in subjects under a constant routine [5], which are: high nocturnal values, a secondary peak in the postprandial region, and the lowest values before sleep onset (a period known as the “wake maintenance zone” [48], [49]. As previously demonstrated for CBT, the WT rhythm seems to be the result of two sets of influences: endogenous, such as autonomic balance directly controlled by the SCN [50], and exogenous, attributable to variables such as light exposure [18], [51], [52], environmental temperature [21], [53], activity [19] and sleep [54].

Despite the fact that modern humans live most of the time in artificial environments, all masking variables recorded here exhibit daily rhythms. Light exposure presented a maximum value at midday, coinciding with a break at work, as has already been observed by other authors [13], [55]–[59]. In our study, activity and body position exhibited similar patterns, with higher values during the period when the subjects were awake, a slightly postprandial decrease and lower values during the sleep period, coinciding with other reports [12], [60]. With regards to environmental temperature, there are no previously published data on the environmental temperature rhythm to which subjects are exposed under free-living conditions, but our data reflects that this rhythm shows colder temperatures during the night and warmer temperatures during the day, with a slight delay with respect to light pattern.

The WT rhythm displays high values when environmental temperatures are low and low values when environmental temperatures are high consistent with other evidence that WT does not respond passively to environmental temperature but rather is regulated to preserve the CBT and brain temperature rhythms [3].

It has been published that exposure to hot environments during sleep periods (at or above 25°C) reduces the amplitude of the CBT rhythm, as it increases nighttime CBT values [21], [53]. Exposure to colder environments during the night (below 25°C), on the other hand, causes a more pronounced decreased in CBT during sleep [21]. As expected, we found that environmental temperature has a strong influence on WT, but only during daytime, when higher environmental temperatures increase WT values. In addition, more robust WT rhythms are obtained under low environmental temperatures, as opposed to medium or high temperatures. Based on these considerations, exposure to moderately cold environmental temperatures may be advisable at night to allow for heat loss through peripheral skin and to contribute to CBT reduction at the beginning of the night, thus facilitating sleep onset [3], [17], [24].

Bright light during the night reduces the nocturnal increase in WT. This fact is consistent with previously published data [52], [61] showing a lower decrease in CBT in response to bright light exposure at night. In addition, an acute increase in light intensity decreases distal skin temperature [13], [62]. Unlike laboratory conditions, however, ambulatory conditions do not allow for the separate analysis of exposure to diurnal light, being awake and being in a vertical position. This therefore makes multiple demasking procedures desirable.

Moderate levels of physical activity are associated with reduced WT because such activity produces heat that increases CBT [36] and with it peripheral skin vasoconstriction that decreases WT.

The orthostatic reflex is presumably the mechanism responsible for the effect of body position on WT [10]. The vasoconstrictor reflex reduces WT while subjects are standing, and increases it when they lie down as a secondary consequence of the vasodilator reflex gated to compensate for the blood pressure increase [10].

As some authors have demonstrated, sleep and distal skin temperature are closely related. So distal temperature, including WT, increases during sleep, only to decrease during waking hours [8], [12], [17], [24]. However, distal temperature can increase, although with lower values, during rest periods without sleep, probably due to relaxation or a recumbent position [17], [24]. However, around 06:00 h, the temperature values of subjects who are awake become similar to those observed in individuals who are asleep, which indicates that this period coincides with that described as the maximum sleepiness zone [63].

Demasking by categories with the constant routine protocol yielded similar results to those considering multiple intercepts at 20 and 25°C, probably due to the environmental temperature range selected (from 19 to 26°C) for category approximation. All constant routine approximations show similar characteristics to those of the original WT pattern, highlighting the endogenous origin of the WT rhythm.

When the influence of each masking variable on WT is considered individually, there is the possibility that mixed confounding influences from other variables may be at work. Therefore, stepwise multiple regression methods may be of interest in order to unmask WT and other rhythms. The intercepts method has yet to be used with simultaneous multiple regression. However, this method allows us to simulate WT changes in response to environmental variables and circadian patterns of sleep-wake or rest-activity rhythms. This model has revealed the existence of a phase-dependent masking effect for each variable, specifically that: a) high environmental temperatures affect WT during the wake period, but not the sleep period, whereas high and low environmental temperatures respectively increase and decrease CBT during sleep [21], [53]; b) bright light reduces WT from the beginning of the sleep period until noon, as it is the case for distal temperature in accordance with the findings of Kräuchi et al. [5]; c) activity decreases WT throughout the day, except during the wake maintenance zone; d) body position modifies WT, but its effect is restricted to the usual times of the main changes in body position, such as awakening and sleep onset; and e) sleep increases WT, as previously described [8], but its effect is the lowest around 06:00 h, the time of maximum sleepiness. Additionally, it is worth noting that variables other than those considered here could contribute to masking and thus affect the unpurified WT pattern.

Our results point to the potential value of WT rhythm in assessing differences in circadian phases in real life conditions. Despite the very homogeneous subject pool of our study, the demasking procedure revealed significant differences in phasing between two chronotype subgroups characterized by different morningness scores. Nonetheless, validation of WT ambulatory recordings to provide clinically useful circadian phase data must come from comparing results from WT to other phase marker rhythms (CBT, melatonin) while contrasting subjects suffering from a number of circadian abnormalities. For example, by studying patients with problems of depression or sleep quality and timing and by examining the responses of subjects to the disruption of the physiological nexus between internal and external times, as occurs in social jet-lag or shift work.

In conclusion, the stepwise multiple regression method allowed us to reduce the masking influence on WT of all four recorded variables by using the independent term to unmask the endogenous circadian component of the WT circadian rhythm. This rhythm has a strong endogenous component, in spite of the influence of different masking variables, each of which affects WT in a phase-dependent manner. However, further experiments will be required to determine whether WT can be established as a marker rhythm for the circadian system under a normal range of environmental and behavioral situations. A further benefit of this achievement would be the suggestion that the multiple demasking procedure used here could become a useful tool for demasking other rhythmic variables, thus providing a new more readily employed standard for circadian system assessment under normal living conditions.

## References

[1] Buijs RM, Kalsbeek A (2001) Hypothalamic integration of central and peripheral clocks. Nature Rev Neurosci 2: 521–526. 11433377 10.1038/35081582

[2] Stratmann M, Schibler U (2006) Properties, entrainment, and physiological functions of mammalian peripheral oscillators. J Biol Rhythms 21: 494–506. 17107939 10.1177/0748730406293889

[3] Van Someren EJW (2000) More than a marker: interaction between the circadian regulation of temperature and sleep, age-related changes, and treatment possibilities. Chronobiol Int 17: 313–354. 10841209 10.1081/cbi-100101050

[4] Mormont MC, Langouët AM, Claustrat B, Bogdan A, Marion S, et al (2002) Marker rhythms of circadian system function: a study of patients with metastatic colorectal cancer and good performance status. Chronobiol Int 19: 141–155. 11962672 10.1081/cbi-120002593

[5] Kräuchi K (2007) The human sleep–wake cycle reconsidered from a thermoregulatory point of view. Physiol Behav 90: 236–245. 17049364 10.1016/j.physbeh.2006.09.005

[6] Aschoff J (1983) Circadian control of body temperature. J Therm Biol 8: 143–147.

[7] Gradisar M, Lack L (2004) Relationships between the circadian rhythms of finger temperature, core temperature, sleep latency, and subjective sleepiness. J Biol Rhythms 19: 157–163. 15038855 10.1177/0748730403261560

[8] Sarabia JA, Rol MA, Mendiola P, Madrid JA (2008) Circadian rhythm of wrist temperature in normal-living subjects: A candidate of new index of the circadian system. Physiol Behav 95: 570–580. 18761026 10.1016/j.physbeh.2008.08.005

[9] Anders D, Vollenweider S, Cann J, Hofstetter M, Flammer J, et al (2010) Heart-rate variability in women during 40-hour prolonged wakefulness. Chronobiol Int 27: 1609–1628. 20854138 10.3109/07420528.2010.504317

[10] Blazquez A, Martinez-Nicolas A, Salazar FJ, Rol MA, Madrid JA (2012) Wrist skin temperature, motor activity and body position as determinants of the circadian pattern of blood pressure. Chronobiol Int 29: 747–756. 22734575 10.3109/07420528.2012.679328

[11] Gomper B, Bromundt V, Orgül S, Flammer J, Kräuchi K (2010) Phase relationship between skin temperature and sleep-wake rhythms in women with vascular dysregulation and controls under real-life conditions. Chronobiol Int 27: 1778–1796. 20969523 10.3109/07420528.2010.520786

[12] Ortiz-Tudela E, Martinez-Nicolas A, Campos M, Rol MA, Madrid JA (2010) A new integrated variable based on thermometry, actimetry and body position (TAP) to evaluate circadian system status in humans. PLoS Comp Biol 6: e1000996 doi:10.1371/journal.pcbi.1000996. 10.1371/journal.pcbi.1000996 PMC2978699 21085644

[13] Martinez-Nicolas A, Ortiz-Tudela E, Madrid JA, Rol MA (2011) Crosstalk between environmental light and internal time in humans. Chronobiol Int 28: 617–629. 21793693 10.3109/07420528.2011.593278

[14] Raymann RJEM, Swaab DF, Van Someren EJW (2008) Skin deep: enhanced sleep depth by cutaneous temperature manipulation. Brain 131: 500–513. 18192289 10.1093/brain/awm315

[15] Romejin N, Van Someren EJW (2011) Correlated fluctuations of daytime skin temperature and vigilance. J Biol Rhythms 26: 68–77. 21252367 10.1177/0748730410391894

[16] Zornoza-Moreno M, Fuentes-Hernandez S, Sanchez-Solis M, Rol MA, Larque E, et al (2011) Assessment of circadian rhythms of both skin temperature and motor activity in infants during the first 6 months of life. Chronobiol Int 28: 330–337. 21539424 10.3109/07420528.2011.565895

[17] Kräuchi K, Wirz-Justice A (2001) Circadian clues to sleep onset mechanisms. Neuropsychopharmacology 25: S92–S96. 11682282 10.1016/S0893-133X(01)00315-3

[18] Cajochen C, Zeitzer JM, Czeisler CA, Dijk DJ (2000) Dose-response relationship for light intensity and ocular and electroencephalographic correlates of human alertness. Behav Brain Res 115: 75–83. 10996410 10.1016/s0166-4328(00)00236-9

[19] Reilly T, Waterhouse J (2009) Circadian aspects of body temperature regulation in exercise. J Thermal Biol 34: 161–170.

[20] Scheer F, Doornen L, Buijs R (1999) Light and diurnal cycle affect human heart rate: Possible role for the circadian pacemaker. J Biol Rhythms 14: 202–212. 10452332 10.1177/074873099129000614

[21] Wakamura T, Tokura H (2002) Circadian rhythm of rectal temperature in humans under different ambient temperature cycles. J Thermal Biol 27: 439–447.

[22] Waterhouse J, Edwards B, Mugarza J, Flemming R, Minors D, et al (1999) Purification of masked temperature data from humans: some preliminary observations on a comparison of the use of an activity diary, wrist actimetry, and heart rate monitoring. Chronobiol Int 16: 461–475. 10442240 10.3109/07420529908998721

[23] Kräuchi K, Cajochen C, Wirz-Justice A (2005) Thermophysiologic aspects of the three process model of sleepiness regulation. Clin Sports Med 24: 287–300. 15892924 10.1016/j.csm.2004.12.009

[24] Kräuchi K, Deboer T (2010) The interrelationship between sleep regulation and thermoregulation. Front Biosci 15: 604–625. 10.2741/3636 20036836

[25] Moore RY, Danchenko RL (2002) Paraventricular-subparaventricular hypothalamic lesions selectively affect circadian function. Chronobiol Int 19: 345–360. 12025929 10.1081/cbi-120002876

[26] Minors D, Waterhouse J (1989) Masking in humans: the problem and some attempts to solve it. Chronobiol Int 6: 29–53. 2650894 10.3109/07420528909059140

[27] Waterhouse J, Weinert D, Minors D, Folkard S, Owens D, et al (2000) A comparison of some different methods for purifying core temperature data from humans. Chronobiol Int 17: 539–566. 10908129 10.1081/cbi-100101063

[28] Weinert D, Waterhouse J (2007) The circadian rhythm of core temperature: Effects of physical activity and aging. Physiol Behav 90: 246–256. 17069866 10.1016/j.physbeh.2006.09.003

[29] Shechter A, Boudreau P, Varin F, Boivin DB (2011) Predominance of distal skin temperature changes at sleep onset across menstrual and circadian phases. J Biol Rhythms 26: 260–270. 21628553 10.1177/0748730411404677

[30] Duffy JF, Dijk DJ (2002) Getting through to circadian oscillators: why use constant routines? J Biol Rhythms 17: 4–13. 11837947 10.1177/074873002129002294

[31] Mills JN, Minors D, Waterhouse J (1978) Adaptation to abrupt time shifts of the oscillator[s] controlling human circadian rhythms. J Physiol 285: 455–470. 745108 10.1113/jphysiol.1978.sp012582 PMC1281767

[32] Rietveld W, Minors D, Waterhouse J (1993) Circadian rhythms and masking: an overview. Chronobiol Int 10: 306–312. 8403074 10.1080/07420529309059713

[33] Reiter RJ, Tan DX, Korkmaz A, Erren TC, Piekarski C, et al (2007) Light at Night, Chronodisruption, Melatonin Suppression, and Cancer Risk: A Review. Crit Rev Oncog 13: 303–328. 18540832 10.1615/critrevoncog.v13.i4.30

[34] Czeisler CA, Duffy JF, Shanahan TL, Brown EN, Mitchell JF, et al (1999) Stability, precision, and near-24-hour period of the human circadian pacemaker. Science 284: 2177–2181. 10381883 10.1126/science.284.5423.2177

[35] Minors D, Waterhouse J (1992) Investigating the endogenous component of human circadian rhythms: a review of some simple alternatives to constant routines. Chronobiol Int 9: 55–78. 1555262 10.3109/07420529209064516

[36] Weinert D, Waterhouse J (1998) Diurnally changing effects of locomotor activity on body temperature in laboratory mice. Physiol Behav 63: 837–843. 9618007 10.1016/s0031-9384(97)00546-5

[37] Waterhouse J, Nevill A, Weinert D, Folkard S, Minors D, et al (2001) Modeling the effect of spontaneous activity on core temperature in healthy human subjects. Biol Rhythm Res 32: 511–528.

[38] Weinert D, Nevill A, Weinandy R, Waterhouse J (2003) The development of new purification methods to assess the circadian rhythm of body temperature in Mongolian gerbils. Chronobiol Int 20: 249–270. 12723884 10.1081/cbi-120018649

[39] Klerman EB, Lee Y, Czeisler CA, Kronauer R (1999) Linear demasking techniques are unreliable for estimating the circadian phase of ambulatory temperature data. J Biol Rhythms 14: 260–274. 10447306 10.1177/074873099129000678

[40] Waterhouse J, Kao S, Weinert D, Edwards B, Atkinson G, et al (2005) Measuring phase shifts in humans following a simulated time-zone transition: agreement between constant routine and purification methods. Chronobiol Int 22: 829–858. 16298771 10.1080/07420520500263375

[41] Weather station of Murcia University's. https://estacion.um.es/.

[42] Horne JA, Östberg O (1976) A self-assessment questionnaire to determine morningness-eveningness in human circadian rhythms. Int J Chronobiol 4: 97–110. 1027738

[43] Portaluppi F, Smolensky MH, Touitou Y (2010) Ethics and methods for biological rhythm research on animals and human beings. Chronobiol Int 25: 1911–1929. 10.3109/07420528.2010.516381 20969531

[44] Van Marken Lichtenbelt WD, Daanen HA, Wouters L, Fronczek R, Raymann RJ, et al (2006) Evaluation of wireless determination of skin temperature using iButtons. Physiol Behav 88: 489–497. 16797616 10.1016/j.physbeh.2006.04.026

[45] Graw P, Haug HJ, Leonhardt G, Wirz-Justice A (1998) Sleep deprivation response in seasonal affective disorder during 40-h constant routine. J Affect Disord. 48: 69–74. 10.1016/s0165-0327(97)00142-0 9495604

[46] Jasper I, Roenneberg T, Häubler A, Zierdt A, Marguardt C, et al (2010) Circadian rhythm in force tracking and in dual task costs. Chronobiol Int 27: 653–673. 20524807 10.3109/07420521003663793

[47] Cajochen C, Knoblauch V, Kräuchi K, Renz C, Wirz-Justice A (2001) Dynamics of frontal EEG activity, sleepiness and body temperature under high and low sleep pressure. Neuroreport 12: 2277–2281. 11447349 10.1097/00001756-200107200-00046

[48] Lavie P (1985) Ultradian rhythms: Gates of sleep and wakefulness. Exp Brain Res 12: 148–164.

[49] Münch M, Knoblauch V, Blatter K, Schröder C, Schnitzler C, et al (2005) Age-related attenuation of the evening circadian arousal signal in humans. Neurobiol Aging 26: 1307–1319. 16182904 10.1016/j.neurobiolaging.2005.03.004

[50] Buijs RM, la Fleur SE, Wortel J, Van Heyningen C, Zuiddam L, et al (2003) The suprachiasmatic nucleus balances sympathetic and parasympathetic output to peripheral organs through separate preautonomic neurons. J Comp Neurol 464: 36–48. 12866127 10.1002/cne.10765

[51] Cajochen C (2007) Alerting effects of light. Sleep Med Rev 11: 453–464. 17936041 10.1016/j.smrv.2007.07.009

[52] Rüger M, Gordijn MCM, Beersma DGM, de Vries B, Daan S (2006) Time-of-day-dependent effects of bright light exposure on human psychophysiology: comparison of daytime and nighttime exposure. Am J Physiol Regul Integr Comp Physiol 290: R1413–R1420. 16373441 10.1152/ajpregu.00121.2005

[53] Kondo M, Tokura H, Wakamura T, Hyun KJ, Tamotsu S, et al (2007) Physiological Significance of Cyclic Changes in Room Temperature around Dusk and Dawn for Circadian Rhythms of Core and Skin Temperature, Urinary 6-hydroxymelatonin Sulfate, and Waking Sensation just after Rising. J Physiol Anthropol 26: 429–436. 17704620 10.2114/jpa2.26.429

[54] Franken P, Tobler I, Borbély A (1992) Sleep and waking have a major effect on the 24-hr rhythm of cortical temperature in the rat. J Biol Rhythms 7: 341–352. 1286205 10.1177/074873049200700407

[55] Goulet G, Mongrain V, Desrosiers C, Paquet J, Dumont M (2007) Daily light exposure in morning-type and evening-type individuals. J Biol Rhythms 22: 151–158. 17440216 10.1177/0748730406297780

[56] Hebert M, Dumont M, Paquet J (1998) Seasonal and diurnal patterns of human illumination under natural conditions. Chronobiol Int 15: 59–70. 9493715 10.3109/07420529808998670

[57] Heil DP, Mathis SR (2002) Characterizing free-living light exposure using a wrist-worn light monitor. Appl Ergon 33: 357–363. 12160339 10.1016/s0003-6870(02)00007-8

[58] Okudaira N, Kripke DF, Webster JB (1983) Naturalistic studies of human light exposure. Am J Physiol Regul Integr Comp Physiol 245: R613–R615. 10.1152/ajpregu.1983.245.4.R613 6624956

[59] Savides TJ, Messin S, Senger C, Kripke D (1986) Natural light exposure of young adults. Physiol Behav 38: 571–574. 3823171 10.1016/0031-9384(86)90427-0

[60] Huang YL, Liu RY, Wang QS, Van Someren EJW, Xu H, et al (2002) Age-associated difference in circadian sleep-wake and rest-activity rhythms. Physiol Behav 76: 597–603. 12126998 10.1016/s0031-9384(02)00733-3

[61] Kim HE, Tokura H (2007) Influence of two different light intensities from 16:00 to 20:30 hours on evening dressing behavior in the cold. Collegium Anthropologicum 31: 145–151. 17598393

[62] Cajochen C, Münch M, Kobialka S, Kräuchi K, Steiner R, et al (2005) High sensitivity of human melatonin, alertness, thermoregulation, and heart rate to short wavelength light. J Clin Endocrinol Metab 90: 1311–1316. 15585546 10.1210/jc.2004-0957

[63] Kräuchi K, Cajochen C, Werth E, Wirz-Justice A (2000) Functional link between distal vasodilation and sleep-onset latency? Am J Physiol Regulatory Integrative Comp Physiol 278: 741–748. 10.1152/ajpregu.2000.278.3.R741 10712296

